# Supplementary material for: Splice-Junction-Based Mapping of Alternative Isoforms in the Human Proteome
Source: Cell Rep. Author manuscript; Available in PMC 2020 Jan 15. (PMC6961840; doi:10.1016/j.celrep.2019.11.026)
Supplement: 3 [file NIHMS1546469-supplement-3.zip › DF2/PXD0010154/Prostate-148-Q96Q05-HRPGALTTNGINPDTSTEIGR.pdf]

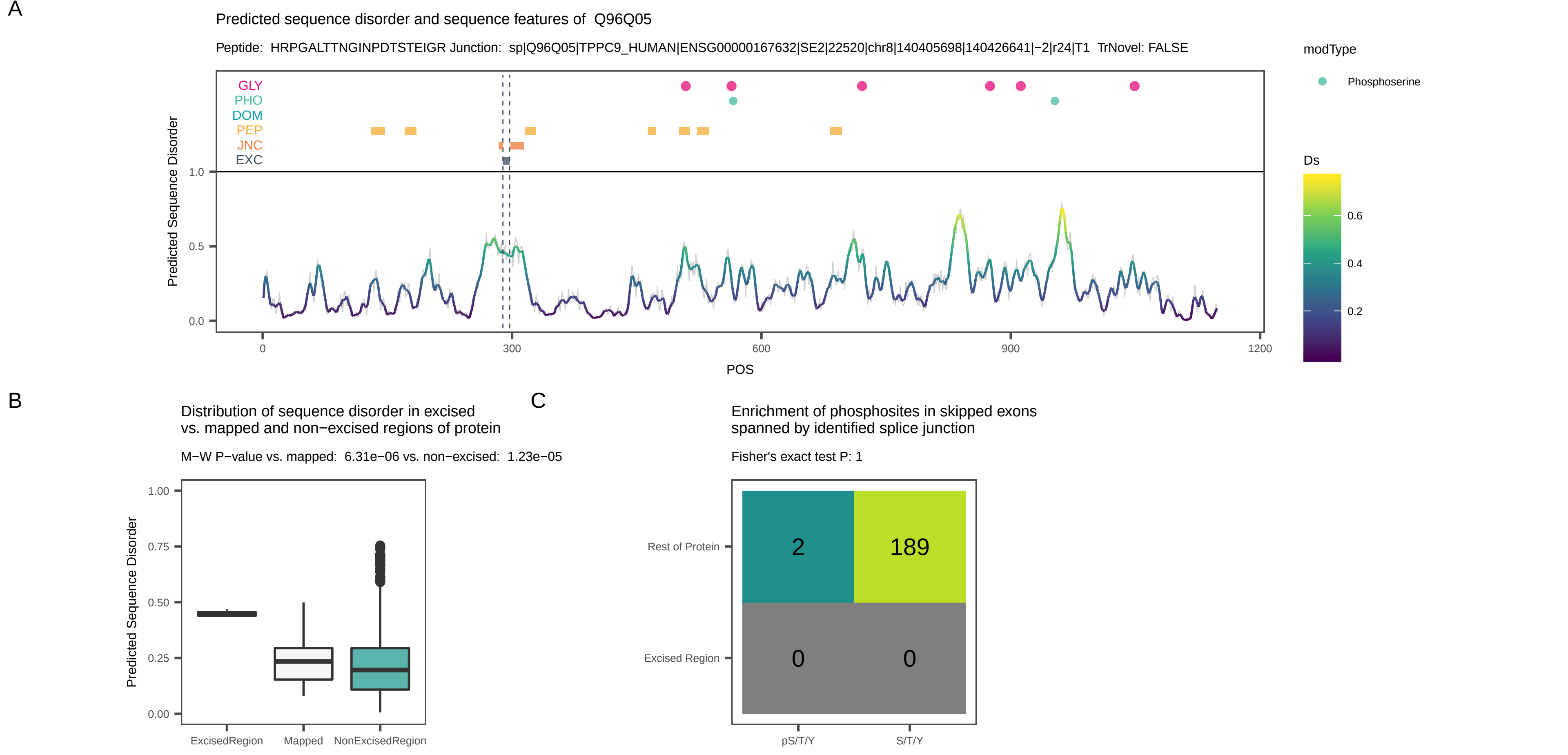
A
Predicted sequence disorder and sequence features of Q96Q05
Peptide: HRPGALTTNGINPDTSTEIGR Junction: sp|Q96Q05|TPPC9_HUMAN|ENSG00000167632|SE2|22520|chr8|140405698|140426641|−2|r24|T1 TrNovel: FALSE
modType
Phosphoserine
GLY
PHO
DOM
PEP
JNC
EXC
Predicted Sequence Disorder
Ds
POS
B
Distribution of sequence disorder in excised vs. mapped and non−excised regions of protein
M−W P−value vs. mapped: 6.31e−06 vs. non−excised: 1.23e−05
Predicted Sequence Disorder
ExcisedRegion
Mapped
NonExcisedRegion
C
Enrichment of phosphosites in skipped exons spanned by identified splice junction
Fisher's exact test P: 1
Rest of Protein
Excised Region
2
189
0
0
pS/T/Y
S/T/Y
